# Integrated Metabolomic and Transcriptomic Analysis of Acute Kidney Injury Caused by *Leptospira* Infection

**DOI:** 10.3390/pathogens11070764

**Published:** 2022-07-04

**Authors:** Kuan-Hsing Chen, Li-Fang Chou, Cheng-Chieh Hung, Hsiang-Yu Tang, Mei-Ling Cheng, Huang-Yu Yang, Hsiang-Hao Hsu, Ya-Chung Tian, Chih-Wei Yang

**Affiliations:** 1 Kidney Research Center, Chang Gung Memorial Hospital, College of Medicine, Chang Gung University, Taoyuan 333, Taiwan; guanhsing3795@gmail.com (K.-H.C.); d928209@gmail.com (L.-F.C.); cchung9651@yhoo.com.tw (C.-C.H.); hyyang01@gmail.com (H.-Y.Y.); hsianghao@gmail.com (H.-H.H.); dryctian@cgmh.org.tw (Y.-C.T.); 2 Healthy Aging Research Center, Chang Gung University, Taoyuan 333, Taiwan; tangshyu@gmail.com (H.-Y.T.); chengm@mail.cgu.edu.tw (M.-L.C.); 3 Metabolomics Core Laboratory, Chang Gung University, Taoyuan 333, Taiwan; 4 Department of Biomedical Sciences, College of Medicine, Chang Gung University, Taoyuan 333, Taiwan; 5 Clinical Phenome Center, Chang Gung Memorial Hospital, Linkou, Taoyuan 333, Taiwan

**Keywords:** leptospirosis, metabolomic, transcriptomic, acute kidney injury, ingenuity pathway analysis (IPA)

## Abstract

Renal leptospirosis caused by leptospiral infection is characterised by tubulointerstitial nephritis and tubular dysfunction, resulting in acute and chronic kidney injury. Metabolomic and transcriptomic data from a murine model of *Leptospira* infection were analysed to determine whether metabolomic data from urine were associated with transcriptome changes relevant to kidney injury caused by *Leptospira* infection. Our findings revealed that 37 metabolites from the urine of *L. interrogans*-infected mice had significantly different concentrations than *L. biflexa*-infected and non-infected control mice. Of these, urinary L-carnitine and acetyl-L-carnitine levels were remarkably elevated in *L. interrogans*-infected mice. Using an integrated pathway analysis, we found that L-carnitine and acetyl-L-carnitine were involved in metabolic pathways such as fatty acid activation, the mitochondrial L-carnitine shuttle pathway, and triacylglycerol biosynthesis that were enriched in the renal tissues of the *L. interrogans*-infected mice. This study highlights that L-carnitine and acetyl-L-carnitine are implicated in leptospiral infection-induced kidney injury, suggesting their potential as metabolic modulators.

## 1. Introduction

Leptospirosis, a zoonotic infectious disease, is caused by pathogenic *Leptospira* spp. infection and is more common in rainy tropical regions. It is a re-emerging global public health issue with increasing occurrence [1]. Leptospirosis-related acute kidney injury (AKI) is characterised by tubulointerstitial nephritis and tubular dysfunction [2,3]. In mice with chronic leptospiral infection, pathogenic *Leptospira* spp. colonise and stay in kidney proximal tubules, causing renal damages in mice [4]. In a recent study, we performed a study with multi-stage sampling and found that leptospiral infection may be related with chronic kidney disease (CKD) in humans [5]. However, the mechanism underlying leptospiral infection-induced tubulointerstitial nephritis and renal fibrosis is still to be elucidated.

Mice have been used in numerous investigations on leptospirosis-associated interstitial nephritis and chronic leptospiral infection [6]. In mice with leptospiral infection, activation of Toll-like receptors (TLRs), nitric oxide synthase, and Na/K-ATPase have all been implicated [7,8,9]. According to in vitro studies, pathogenic *Leptospira* spp. may be able to evade innate immune responses to infection by resisting the complement system [10]. Significant alterations in the transcript levels of genes associated with cytokines/chemokines have also been discovered in kidney tissues of leptospiral-infected animals [11]. The changes of the microenvironment in kidney injury caused by pathogenic *Leptospira* spp. infection could be explained by the activation of cytokine/chemokine cascades. According to our previous study on renal transcriptome changes caused by leptospiral infection, pathways such as the complement system, immunological function, and the interactions between cells were significantly enriched in pathogenic *L. interrogans*-infected mice [12]. Importantly, bacterial adherence to the tubule lumen was found in the kidneys of pathogenic *L. interrogans*-infected mice, but not in the kidneys of nonpathogenic *L. biflexa*-infected mice [12]. Our findings provide a comprehensive understanding of the transcriptional profiles in murine kidneys after leptospiral infection.

Metabolomics is an approach for evaluating in vivo metabolite profiles that is both comprehensive and systemic. To date, there have been no studies investigating the effects of leptospiral infection on urinary metabolomics in AKI. To understand the association between changes in urinary metabolomics and the transcriptome in pathogenic and nonpathogenic *Leptospira* spp., we performed non-targeted urinary metabolomics and ingenuity pathway analysis (IPA) in this study. We found that urinary metabolites, such as L-carnitine and acetyl-L-carnitine, are involved in pathways associated with energy metabolism and are enriched in renal tissues in pathogenic leptospiral-infected mice. Integrated metabolomic and transcriptomic analyses revealed that a disturbance in energy metabolism is essential in developing AKI caused by pathogenic *Leptospira* spp. infection.

## 2. Results

### 2.1. Characterisation of Urine Metabolite Trends in Leptospiral-Infected Mice Using LC-TOFMS-Based Metabolomics

**Figure 1:**
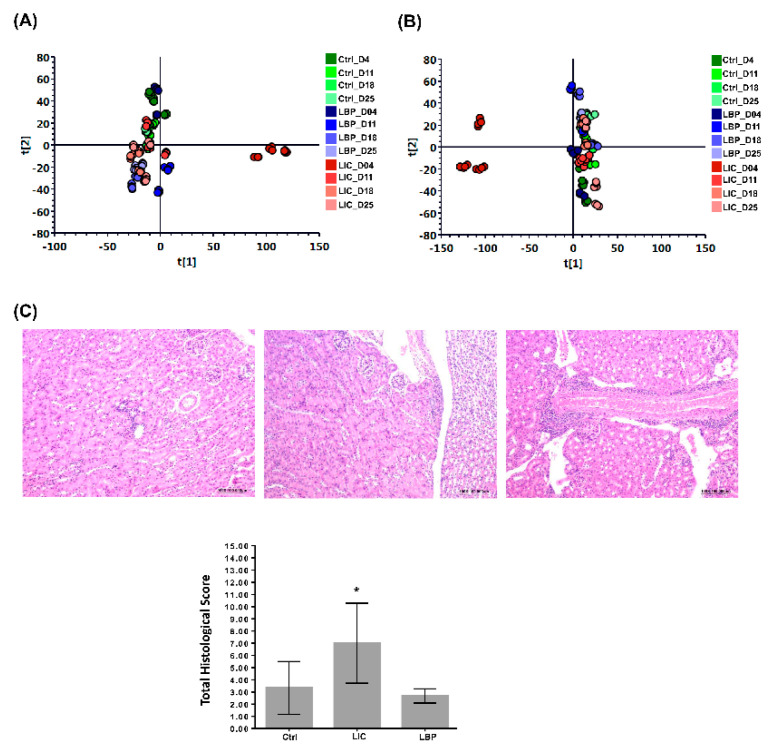
Orthogonal partial least squares discriminant analysis (OPLS-DA) of the urinary metabolomes of non-infected (Ctrl) mice, mice infected with nonpathogenic *L. biflexa* serovar Patoc (LBP), and mice infected with pathogenic *L. interrogans* serovar Copenhageni (LIC) at different time points, as well as renal histopathological analysis of the mice in the different groups at day 7 post-infection. (**A**) OPLS-DA of urine metabolomes obtained in the ESI+ mode. (**B**) OPLS-DA of urine metabolomes obtained in the ESI− mode. (**C**) Representative HE staining of the tubulointerstitial tissue of mice in the different groups. The corresponding histological scores are shown. *: denotes a significant difference between LIC with Ctrl or LBP group (*p* < 0.05).

To generate urine metabolomes in non-infected and infected mice with nonpathogenic *L. biflexa* or pathogenic *L. interrogans*, a global metabolite study employing LC-MS was combined with univariate and multivariate statistical analyses. The OPLS-DA was further analysed to understand the relationship between urinary metabolites at different time points. The OPLS-DA score plots of the ESI+ and ESI− modes (Figure 1A,B, respectively) show that the clusters in the non-infected and nonpathogenic *Leptospira* spp.-infected groups at different time points were quite close, but the clusters in the pathogenic *Leptospira* spp.-infected group during the first week (LIC_D04) were well discriminated from the others. These results indicate that the urinary metabolite profiles were significantly different in the first week after pathogenic *Leptospira* spp. infection. Therefore, in the following experiments, we focused on changes in urine metabolomics and renal genetic analysis in the first week after pathogenic *Leptospira* spp. infection.

### 2.2. Pathological Analysis of Renal Injury in the Different Groups of Mice at Day 7

The results of the histological evaluation of AKI in mice are shown in Table 1. Selected pathological images of the different groups are shown (Figure 1C). The degree of total histopathological lesions (inflammatory cell infiltration, tubular lumen degeneration/necrosis, tubular lumen dilation, and fibrosis) in the mouse kidneys after infection with pathogenic *Leptospira* spp. at day 7 increased significantly when compared to the control mice, according to statistical analysis (*p* < 0.05) (Figure 1C). Our histopathological data indicate that mice infected with pathogenic *Leptospira* spp. appeared to have more severe kidney injuries on day 7.

### 2.3. Urinary Metabolites in Leptospira spp.-Infected Mice in the First Week

**Figure 2:**
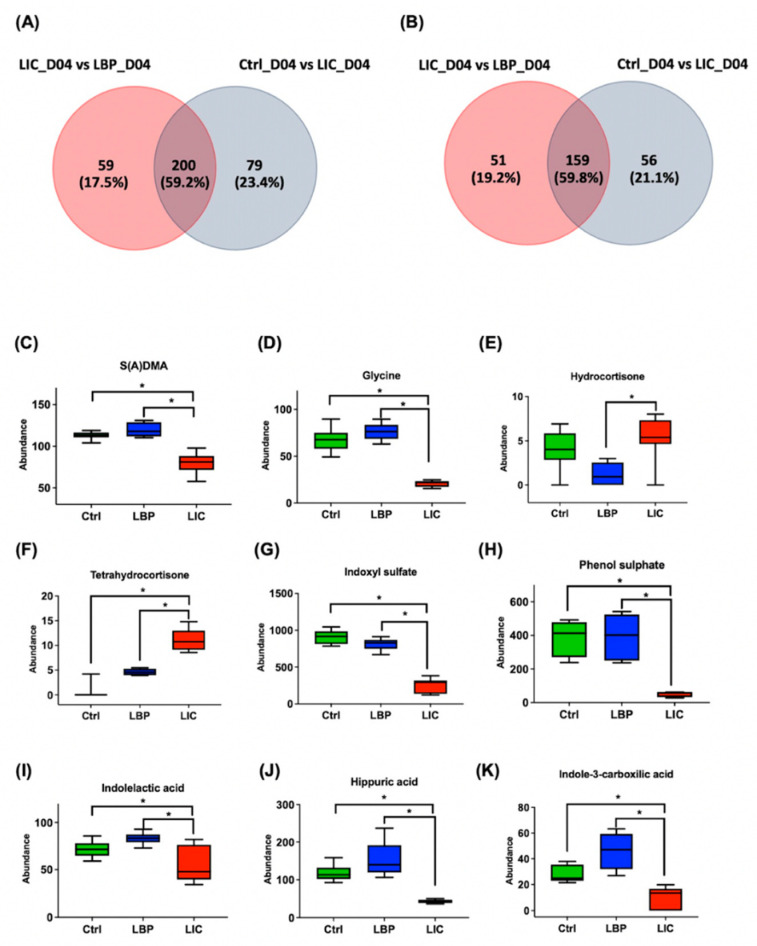
Urine metabolomes of non-infected (Ctrl) mice, mice infected with nonpathogenic *L. biflexa* serovar Patoc (LBP), and mice infected with pathogenic *L. interrogans* serovar Copenhageni (LIC) on day 4 post-infection. (**A**) Venn diagram of the number of metabolites with VIP > 1 and significant significance (*p* < 0.05) between the three groups as obtained in ESI+ mode. (**B**) Venn diagram of the number of metabolites with VIP >1 and significant significance (*p* < 0.05) between the three groups as obtained in ESI− mode. Also shown are box-and-whisker plots of the significantly different urinary metabolites amongst the three groups. The integrated intensities of S(A)DMA (**C**), glycine (**D**), hydrocortisone (**E**), tetrahydrocortisone (**F**), indoxyl sulphate (**G**), phenol sulphate (**H**), indolelactic acid (**I**), hippuric acid (**J**), and indole-3-carboxylic acid (**K**). *: denotes a significant difference between groups (*p* < 0.05).

**Figure 3:**
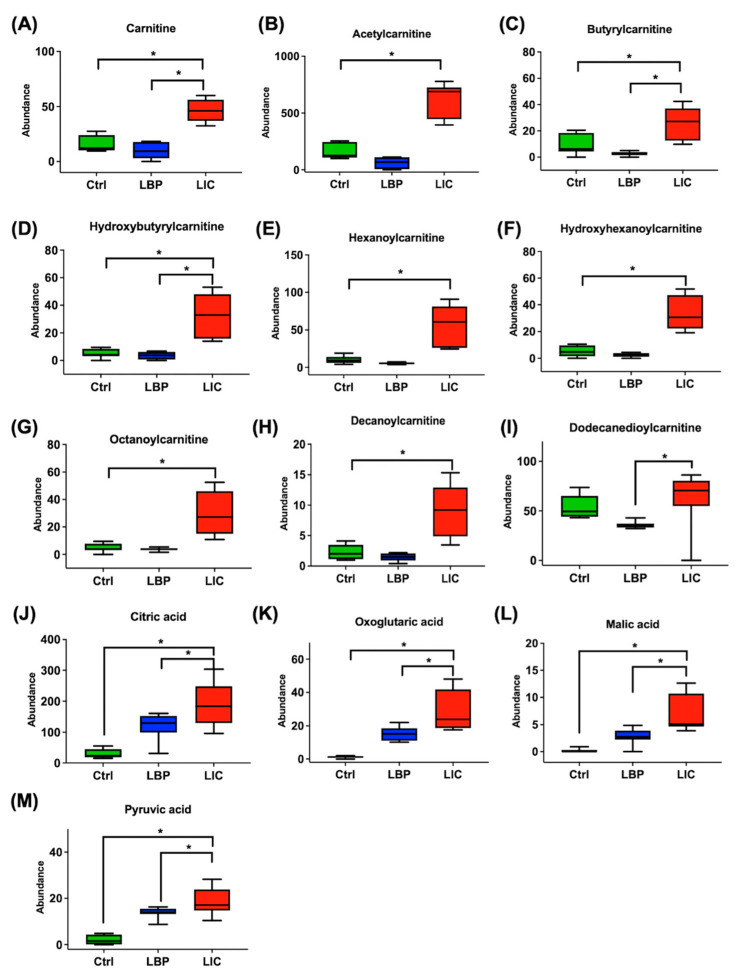
Box-and-whisker plot of the urinary metabolites with significant differences amongst the non-infected (Ctrl) mice, mice infected with nonpathogenic *L. biflexa* serovar Patoc (LBP), and mice infected with pathogenic *L. interrogans* serovar Copenhageni (LIC) on day 4 post-infection. The integrated intensities of carnitine (**A**), acetylcarnitine (**B**), butyrylcarnitine (**C**), hydroxybytyrylcarnitine (**D**), hexanoylcarnitine (**E**), hydroxyhexanoylcarnitine (**F**), octanoylcarnitine (**G**), decanoylcarnitine (**H**), dodecanedioylcarnitine (**I**), citric acid (**J**), oxoglutaric acid (**K**), malic acid (**L**), and pyruvic acid (**M**) are as shown. The bottom and top of the boxes indicate the 25th and 75th percentiles, respectively, while the whiskers indicate the 5th and 95th percentiles, respectively. The *p*-values were calculated using ANOVA with Tukey HSD correction. *: denotes a significant difference between groups (*p* < 0.05).

Metabolites with substantial-level differences (VIP score > 1) amongst the three groups were further investigated. Metabolites at the intersection between the three groups were selected as the significant metabolites of the group of mice infected with pathogenic *Leptospira* spp. In the ESI+ mode, 200 metabolites were specific to the group of mice with *L. interrogans* infection; meanwhile, in the ESI− mode, there were 159 metabolites unique to *L. interrogans*-infected mice. These metabolites were then searched against the HMDB and KEGG databases. Based on their chromatographic retention times as well as MS and MS/MS data from the standards utilised, a number of metabolites were selected and identified. Figure 2 and Figure 3 show the relative signal intensities of the targeted metabolites in the different groups. In the *L. interrogans*-infected mice, the urinary levels of S(A)DMA (Figure 2C), glycine (Figure 2D), indoxyl sulphate, phenol sulphate, indolelactic acid, hippuric acid, and indole-3-carboxylic acid (Figure 2G–K) were significantly reduced compared to those in non-infected and nonpathogenic *L. biflexa*-infected mice. However, in mice infected with pathogenic leptospires, urinary cortisone levels, such as those of hydrocortisone and tetrahydrocortisone (Figure 2E,F), were significantly higher than in non-infected and nonpathogenic *L. biflexa*-infected mice. In addition, the levels of urinary metabolites associated with energy metabolism (Figure 3A–M), such as carnitine, acyl-carnitines, and TCA cycle metabolites, were also elevated in pathogenic leptospiral-infected mice compared to non-infected and nonpathogenic leptospiral-infected mice.

### 2.4. RNA-Seq-Based Transcriptomics for Functional Pathway Analysis

Illumina RNA-seq was performed to profile renal transcriptomes from mice infected with pathogenic and nonpathogenic *Leptospira* spp. at seven days post-infection. Using differential expression gene (DEG) analysis with an absolute fold-change (FC) ≥ 1.5 and *p* values < 0.05, a total of 1124 DEGs, including 805 upregulated and 319 downregulated DEGs, were obtained between the pathogenic leptospiral-infected and the non-infected groups. Compared with the non-infected group, 109 transcripts (54 upregulated and 55 downregulated) were differentially expressed in the group infected with the nonpathogenic *L. biflexa*.

**Figure 4:**
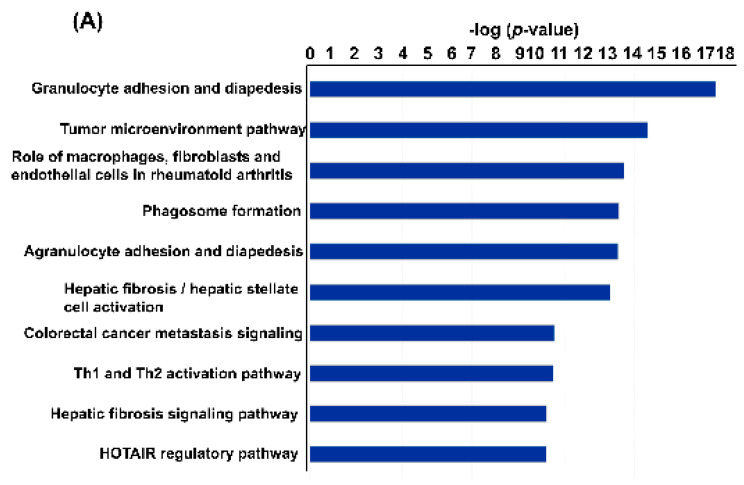

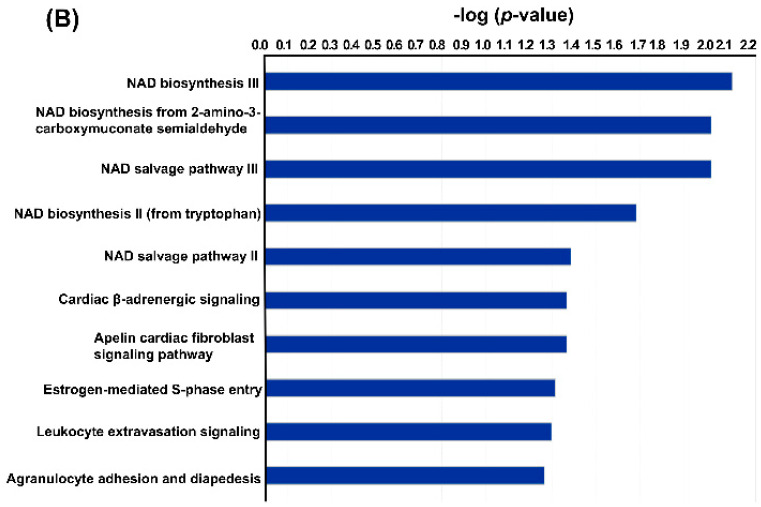
Significantly enriched IPA canonical signalling pathways. Significant canonical pathways are listed and ranked by *p*-value. The vertical axis indicates canonical pathways, while the horizontal axis indicates the −log(*p*-value). The top 10 significantly enriched canonical pathways in the renal transcriptome of mice infected with pathogenic *L. interrogans* (**A**) and nonpathogenic *L. biflexa* (**B**).

**Figure 5:**
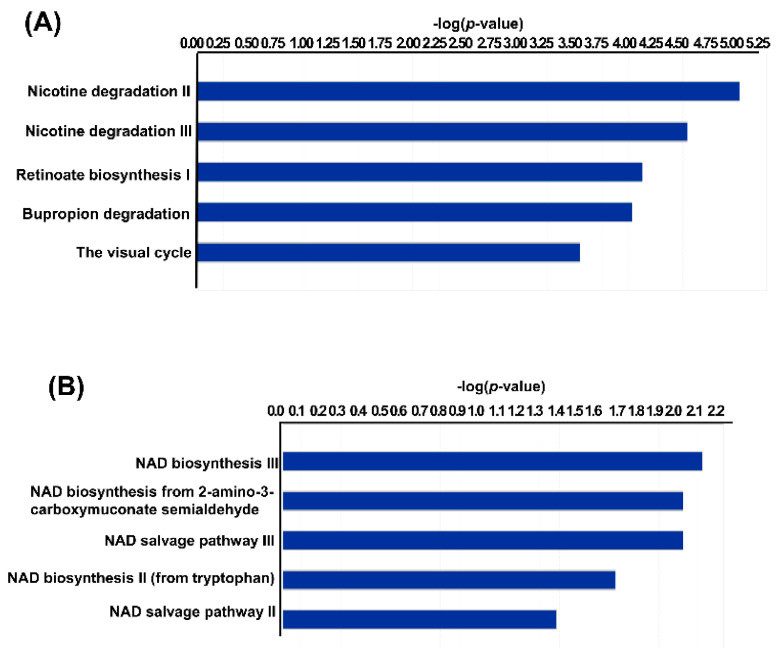
Top five enriched metabolic canonical pathways identified from the renal transcriptome of mice infected with pathogenic *L. interrogans* (**A**) and nonpathogenic *L. biflexa* (**B**). The vertical axis indicates the canonical pathways, while the horizontal axis indicates the −log (*p*-value).

To determine the functional effects of pathogenic and nonpathogenic *Leptospira* spp. infection-induced changes in renal gene expression, the identified DEGs were investigated using IPA with the filtering option *p* < 0.5. Using pathway enrichment analysis, we found that pathogenic *L. interrogans-* and nonpathogenic *L. biflexa*-infected murine kidney tissues had 402 and 92 enriched pathways, respectively (Table S1). Figure 4 depicts the top 10 significantly enriched canonical pathways identified using IPA enrichment analysis of the renal transcriptomes. As indicated in Figure 4A, significant IPA canonical pathways associated with immune function and fibrosis were enriched in the renal transcriptomes of mice with AKI caused by pathogenic *L. interrogans* infection. To determine the top metabolic pathways enriched in each dataset, the data were subjected to IPA core analysis and then analysed using canonical metabolic pathways. Using IPA metabolic pathway analysis, we found that pathogenic *L. interrogans*- and nonpathogenic *L. biflexa*-infected murine kidney tissues had 73 and 6 enriched metabolic pathways, respectively (*p* < 0.5, Table S2). As shown in Figure 5, in pathogenic *L. interrogans* compared to nonpathogenic *L. biflexa*, the top five enriched canonical metabolic pathways identified using IPA analysis of renal transcriptomes were metabolic pathways associated with nicotine degradation and nicotinamide adenine dinucleotide (NAD) biosynthesis.

### 2.5. Integrated Pathway-Level Analysis of Transcriptomic and Metabolomics Data

**Figure 6:**
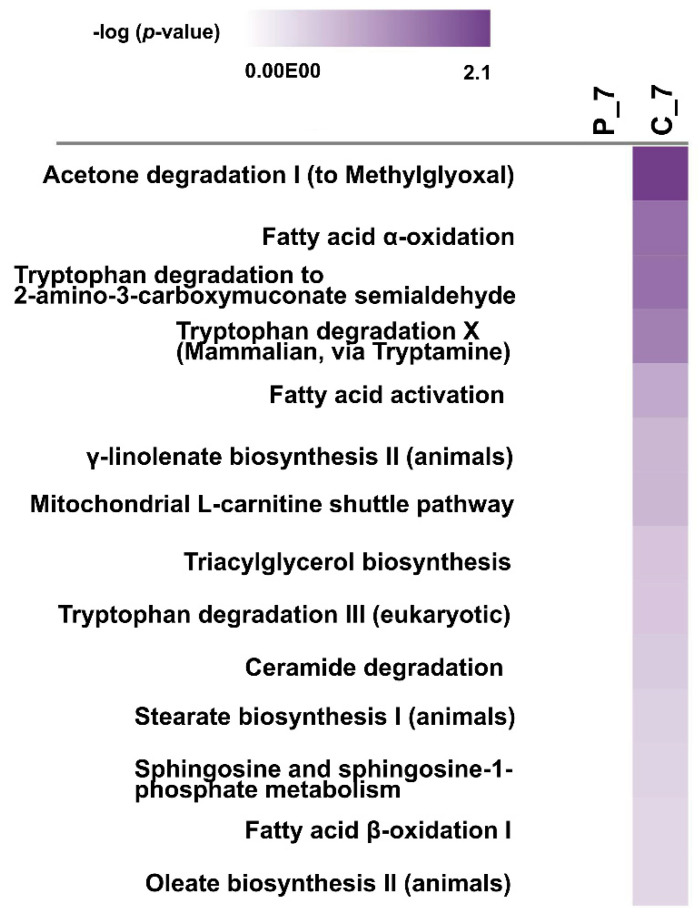
Heat map showing the IPA comparison analysis of the pathogenic *L. interrogans*-infected group and nonpathogenic *L. biflexa*-infected group. The canonical metabolic pathways are ranked by the negative log of the *P*-value of the enrichment score. C_7: renal transcriptome of mice infected with pathogenic *L. interrogans* at day 7; P_7: renal transcriptome of mice infected with nonpathogenic *L. biflexa* at day 7.

A comparison study was performed using the comparison analysis function in IPA to evaluate the differences amongst the enriched metabolic pathways. A comparison of the DEG-driven metabolic pathway analysis between the pathogenic leptospiral-infected group and the nonpathogenic leptospiral-infected group indicated that 419 canonical metabolic pathways were enriched (*p* < 0.5). Of the enriched pathways, 14 were associated with fatty acid activation/biosynthesis/degradation, lipid biosynthesis, and tryptophan biosynthesis and degradation (*p* < 0.5, Table S3 and Figure 6).

**Figure 7:**
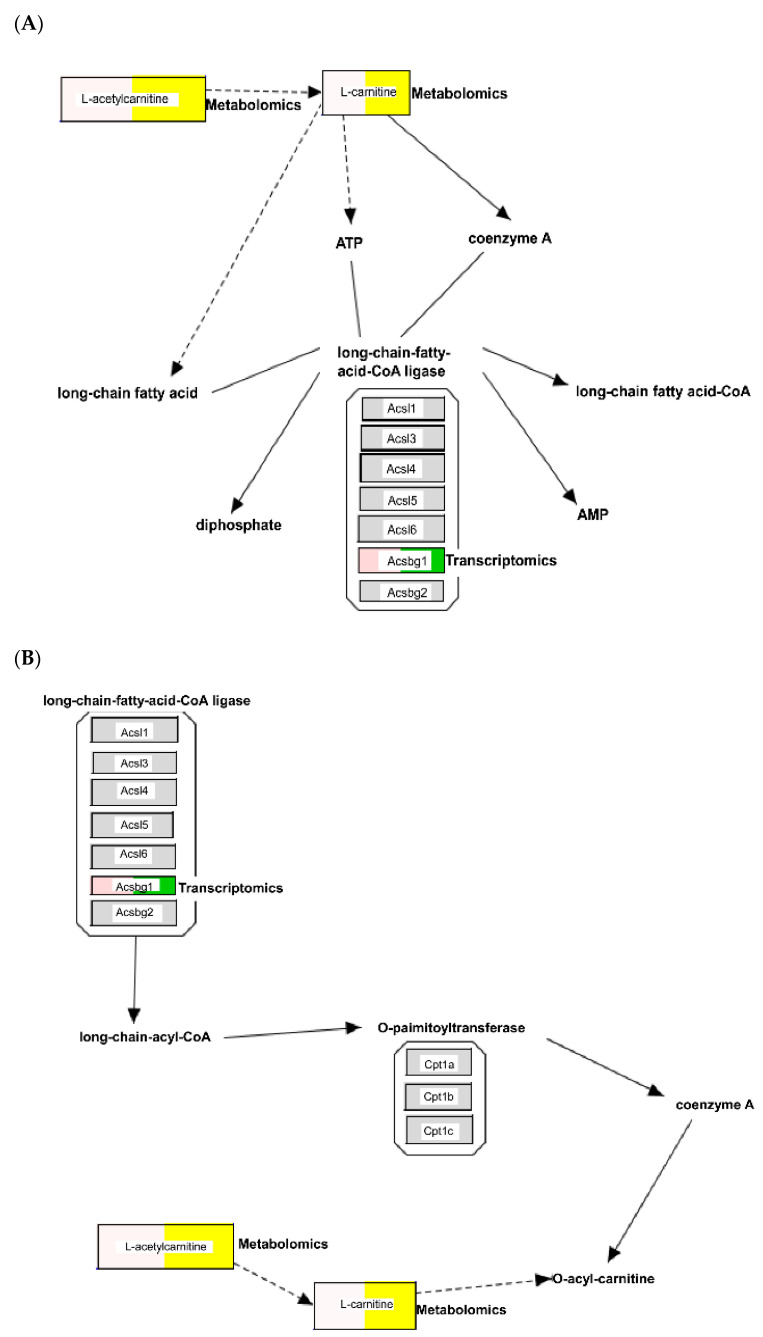

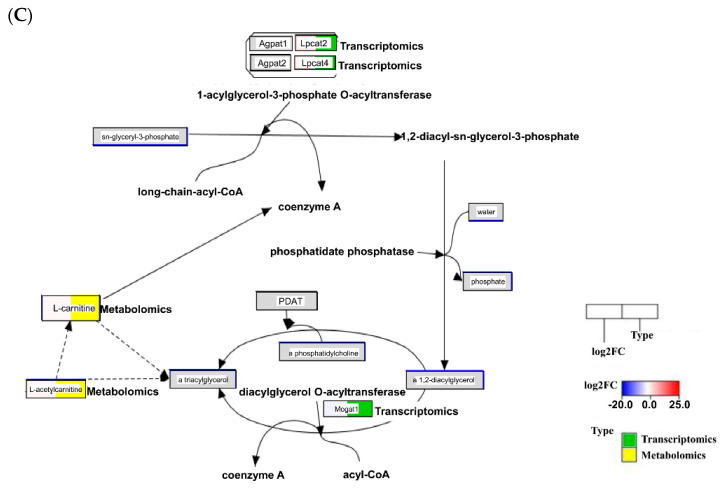
Visualisation of the integrated pathway-level analysis from transcriptomics and metabolomics data using PathVisio. Figures depict the differentially expressed genes (transcriptomics; green) and metabolites (metabolomics; yellow) that participate in the canonical pathways relevant for fatty acid activation (**A**), the mitochondrial L-carnitine shuttle pathway (**B**), and triacylglycerol biosynthesis (**C**). The visualisation of the boxes is split into two parts: (1) the log_2_ FC values are in the left part of the box (the intensity of the blue or red colour), and (2) the types are indicated in the right part of the box (transcriptomics: green, metabolomics: yellow). Pathway elements, including transcripts, that were not changed are coloured grey.

The relationship between metabolomics and transcriptomic data was also queried using IPA, resulting in overlapping direct interaction networks. Forty-six metabolites could be used to discriminate between the urine of pathogenic leptospiral-infected and nonpathogenic leptospiral-infected mice. Of these, thirty-seven metabolites were mapped to their corresponding metabolites in the IPA knowledge base (Table S4). To examine changes in urine metabolites associated with tissue transcriptome differences caused by leptospiral infection, urinary metabolite data (Table S4) were overlaid on the enriched canonical metabolic pathways associated with energy metabolism (Figure 6) using the overlay tool in IPA. Pathway maps integrating the transcriptomic and metabolomic data were visualised using PathVisio [13]. As shown in Figure 7, we observed an increase in the concentrations of L-carnitine and acetyl-L-carnitine in the urine of pathogenic *L. interrogans*-infected mice, which seem to modulate the canonical pathways for fatty acid activation, mitochondrial L-carnitine shuttle pathway, and triacylglycerol biosynthesis.

## 3. Discussion

This is the first study to use metabolomics to investigate the differences between mice with pathogenic and nonpathogenic *Leptospira* spp. infection. From the serial urine metabolomic analysis performed at different time points after infection, we confirmed that only pathogenic *L. interrogans*-infected mice had significantly different urinary metabolite profiles compared to other groups in the first week post-infection. Thus, in the following experiments, we focused on the metabolomic, pathological, and transcriptomic changes in the mice one week after infection.

Pathological analysis revealed that pathogenic *L. interrogans*-infected mice had significantly more focal tubular lumen degeneration/necrosis, multifocal tubular dilation, and focal fibrosis lesions in the kidneys than non-infected and nonpathogenic *Leptospira* spp.-infected mice. These findings indicate acute tubulointerstitial injury in mice one week after infection with pathogenic *L. interrogans*. These results may explain the differences in urinary metabolomics in mice during the first week of leptospiral infection.

To realise the important metabolites related to pathogenic *L. interrogans* infection, we identified the significantly different metabolites in pathogenic *L. interrogans*-infected mice compared to the non-infected and nonpathogenic *Leptospira* spp.-infected mice. Most of the identified metabolites were associated with kidney injury, acute stress, and dysregulated energy metabolism in mice infected with pathogenic *L. interrogans*. The catabolic product of post-translational modifications on methylated arginine-containing proteins, symmetric dimethylarginine (SDMA; MW, 202 g/mol), is primarily removed by the kidneys [14]. SDMA levels in the blood have been demonstrated to increase in patients with renal disease and are correlated with the glomerular filtration rate (GFR) [15]. Therefore, SDMA is a good biomarker that can be used to assess kidney function. In pathogenic *L. interrogans*-infected mice, the urinary level of SDMA was significantly reduced compared to that in non-infected and nonpathogenic *Leptospira* spp.-infected mice. This difference indicates that glomerular filtration decreased in pathogenic *L. interrogans*-infected mice compared to the other groups. In addition, the excretion of uraemic toxin (phenol sulphate) and tryptophan degradation metabolites (indoxyl sulphate, indolelactic acid, and indole-3-carboxylic acid) was significantly reduced in pathogenic *L. interrogans*-infected mice. These metabolites are primarily secreted into the renal tubules by OAT4 on the luminal side of tubular cells [16]. In mice infected with pathogenic *L. interrogans*, the function of OAT4 was impaired due to tubule-interstitial injury, which resulted in the reduced excretion of these metabolites. In pathogenic *L. interrogans*-infected mice, urinary hippuric acid excretion was also reduced; a decrease in urinary hippuric acid excretion has previously been demonstrated in patients with AKI [17].

However, some urine metabolites were significantly higher in pathogenic leptospiral-infected mice compared to those in the non-infected and nonpathogenic leptospiral-infected mice. Increased urinary cortisone levels reflect a systemic stress response to AKI. Therefore, in pathogenic *L. interrogans*-infected mice, the urinary levels of hydrocortisone and tetrahydrocortisone were significantly elevated compared to those in non-infected and nonpathogenic *Leptospira* spp.-infected mice [18]. Similarly, elevated levels of urinary carnitine and acylcarnitines (including acetylcarnitine and other medium-to-long-chain carnitines) were noted in pathogenic *L. interrogans*-infected mice compared to those in non-infected and nonpathogenic *Leptospira* spp.-infected mice. Fatty acids are transported into the cells and utilised for energy production via β-oxidation in the mitochondria. Specifically, fatty acyl groups are transferred across the mitochondrial membrane via the carnitine carrier system for β-oxidation. The expression and activity of mitochondrial fatty acid oxidation enzymes in the kidney tissue have been found to decrease during AKI [19,20]. A previous study found that total carnitine, free carnitine, and short-chain and long-chain acylcarnitine concentrations in plasma were all higher in patients with AKI than in healthy subjects [21]. Therefore, in mice infected with pathogenic *L. interrogans*, elevated urinary carnitine and acylcarnitine levels indicate the dysregulation of energy metabolism in renal tissues due to AKI. Moreover, levels of TCA cycle metabolites (citric acid, oxoglutaric acid, malic acid, and pyruvic acid) were significantly increased in the urine of pathogenic *L. interrogans*-infected mice. Dysregulated energy metabolism and mitochondrial dysfunction in renal tubules have been demonstrated in AKI [22,23]. Changes in the metabolites of TCA cycle intermediates in kidney tissues have been shown in an ischaemic reperfusion AKI model [22]. Our results also confirmed that when mice were infected with pathogenic *L. interrogans*, elevated levels of TCA cycle metabolites in the urine reflected energy dysregulation in *Leptospira*-induced AKI.

According to our RNA-seq-based functional transcriptomic data, nicotine degradation and NAD biosynthesis were associated with murine kidney tissues infected with pathogenic and nonpathogenic *Leptospira* spp., respectively. Nicotine degradation mechanisms have been identified in microorganisms such as *Pseudomonas* spp. and *Arthrobacter* sp. Strain 68b [24,25]. Importantly, a putative nicotine degradation pathway was identified in pathogenic *L. interrogans*. Furthermore, nicotine-induced changes could potentially inhibit the growth of various microorganisms, inducing more intense antigenic stimulations [26]. NAD is a metabolite that plays a vital role in cellular health, affecting DNA repair and the cellular lifespan. In recent investigations, NAD metabolism has been identified as a therapeutic target for metabolic diseases and infections and it has been hypothesised that increasing NAD levels may protect the renal tubules against a variety of acute stresses [27,28].

To further investigate the association between urinary metabolites and gene expression in murine kidneys, we combined metabolomics and transcriptomics data for further analysis. When transcriptomic and metabolomic data are integrated, the association between the metabolites and transcripts cannot be analysed directly; it would be difficult to link each metabolite to a transcript and vice-versa. This is in contrast to transcriptomics and proteomics data, where most transcripts can be mapped to a single protein. However, transcriptomic–metabolomic integration remains a powerful analytical tool since the metabolome provides phenotypic measurements to which we can anchor the global measurements of the transcriptome. According to the IPA results of our transcriptomic and metabolomics data, AKI caused by infection with pathogenic *Leptospira* spp. resulted in significantly higher levels of urinary L-carnitine and acetyl-L-carnitine. This upregulation is linked to the expression of transcripts related to the fatty acid activation pathway, the mitochondrial L-carnitine shuttle pathway, and the triacylglycerol biosynthesis pathway in mouse kidney tissue. These findings indicate the importance of dysregulated fatty acid metabolism and mitochondrial dysfunction in pathogenic *Leptospira* spp.-induced AKI.

Compounds with antioxidant action in the kidneys, such as fatty acid binding protein, are released into urine by injured renal tubules in response to oxidative stress [29]. According to the studies of Alan M. Arau’jo et al., oxidative stress occurred in patients with severe leptospirosis and was linked to renal dysfunction and thrombocytopenia [30]. Importantly, our findings reveal that fatty acid metabolism is linked to AKI induced by leptospiral infection. This may guide the development of novel adjuvant therapies for leptospirosis that target oxidative stress. Moreover, Irene F Gazi et al. investigated the effects of acute infection with *L. interrogans* on lipids and associated enzymes, finding that leptospirosis was associated with markedly increased triglycerides [31]. In our study, the results reveal that triacylglycerol biosynthesis is involved in murine kidney tissues infected with pathogenic *Leptospira* spp. In addition, our study is the first to describe L-carnitine and acetyl-L-carnitine in leptospiral infection-induced kidney injury using a murine infection model, and their relevance in human leptospiral diseases needs to be proven experimentally in the future.

## 4. Materials and Methods

### 4.1. Bacterial Culture

Pathogenic *L. interrogans* serovar Copenhageni Fiocruz L1-130 (ATCC BAA-1198) and nonpathogenic *L. biflexa* serovar Patoc (ATCC 23582) were propagated in a Leptospira medium base Elinghausen-McCullough-Johnson-Harris (EMJM) medium (Difco) including Leptospira enrichment EMJH medium at 28 °C. Dark-field microscopy and a Petroff-Hausser counting chamber were used to determine the number of bacteria in a population.

### 4.2. Mouse Model for Leptospiral Infection

C57BL/6 mice (females) at 7–8 weeks of age were injected intraperitoneally with an infective dose of 10^9^ bacteria cells while the control group was inoculated with sterile EMJH medium [32]. Urine samples from the different groups of mice were collected at 4, 11, 18, and 25 days post-infection for non-targeted metabolomic analysis. Animal biosafety level 2 conditions were required for all animal experiments, as well as adherence to all applicable rules for the use and management of animals. The Institutional Animal Care and Use Committee of Chang Gung Memorial Hospital authorised all animal procedures and experimental methods (no. 2015120101).

### 4.3. Urine Sample Extraction for Metabolomics Analysis

Using distilled water, the urine samples were diluted to a creatinine content of 20 g/mL. For liquid chromatography–mass spectrometry (LC-MS) analysis, the supernatants were collected and centrifuged for 5 min at 14,000× *g*.

### 4.4. Kidney Tissue Preparation

Seven days post-infection, kidneys were collected after the mice were anaesthetised and sacrificed. For RNA analysis, the kidneys were hemi-sectioned and frozen in liquid nitrogen. Some kidneys were fixed for 12 h at 4 °C in 10% neutral buffered formalin, then processed, embedded in paraffin wax, sliced into 4 mm sections, and stored at room temperature (22~25 °C) until examination.

### 4.5. Histopathological Analysis of the Kidneys

To assess kidney structural injury, the hematoxylin and eosin (HE) stain was performed according to the manufacturer’s guidelines. BioLASCO Taiwan Co., Ltd.’s veterinary pathologist performed histological examinations via optical microscope (Leica DM2700M, Wetzlar, Germany). The total histopathological lesions (inflammatory cell infiltration, tubular lumen degeneration/necrosis, tubular lumen dilation and fibrosis) in kidney tissue were assessed. The severity of the histopathological lesions was determined using quantitative recording of histopathological lesions in kidney tissue sections according to Shackelfold et al. [33].

### 4.6. Liquid Chromatography/Time-of-Flight Mass Spectrometry (LC/TOFMS) Analysis

Using a HPLC system (1200 rapid resolution system; Agilent Technologies, Santa Clara, CA, USA), liquid chromatographic separation was obtained on a 100 mm × 2.1 mm Acquity 1.7-m C18 column (Waters Corp, Milford, MA, USA). The flow rate was 0.25 mL/min and the column was kept at 40 °C. The following linear gradients of solvent A (2 mM ammonium formate in water) and solvent B (100% ACN) were used to elute samples from the LC column: −1 min, 0% B; 1–9.6 min, 0–98% B; 9.6–15 min, 98% B; and 15–18 min, 0% B. An Agilent Q-TOFMS (6510 Q-TOFMS; Agilent Technologies, Santa Clara, CA, USA) was used to perform mass spectrometry in both electrospray positive-ion (ESI+) and electrospray negative-ion (ESI-) modes. The scan range was 50–1000 *m*/*z*.

The capillary and skimmer voltages were set at 4000 and 65 V, respectively; the liquid nebuliser was set to 30 psig, the nitrogen drying gas was set at a flow rate of 10 L/min, and the drying gas temperature was maintained at 350 °C. Purine ([M-H] 121.050873), hexakis (1H,1H,3H-tetrafluoropropoxy) phosphazine ([M-H] 922.009798), purine ([M-H] 119.036320), and formate adduct ([M-H] 966.000725) were utilised as internal reference ions to ensure constant mass accuracy. Data were obtained using data acquisition software in profile mode (Agilent MassHunter Workstation, Agilent Technologies, Santa Clara, CA, USA).

### 4.7. Data Processing

A molecular feature extraction algorithm (MassHunter, Agilent Technologies, Santa Clara, CA, USA) was used to extract information about the metabolites of interest from the raw data. In addition to the time-aligned ion characteristics, the algorithm also provided the monoisotopic neutral mass, retention time, and ion abundance. GeneSpring-MS was used to analyse and visualise the patterns of the MassHunter data matrices. Clustering and correlation analyses were performed using orthogonal partial least squares discriminant analysis (OPLS-DA). ANOVA with Tukey’s HSD correction was used to compare the relative concentrations of the metabolites. Multivariate data analysis and representation were performed with the same software. The METLIN, Human Metabolome Database (HMDB), and KEGG databases were searched for accurate masses of characteristics that differed significantly between the test and control groups. The Metabolite Database and Molecular Formula Generation Software (Agilent Technologies, Santa Clara, CA, USA) were used to predict compounds.

### 4.8. Metabolite Identification

We employed the same chromatographic conditions for structural identification as we did for the metabolite standards profiling experiment. The following parameters were used for mass spectrometry with an Agilent 6510 Q-TOFMS: gas temperature, 350 °C; flow rate of drying gas, 10 L/min; nebuliser pressure, 30 psig; capillary voltage, 4000 V; fragmentor voltage, 175 V; and skimmer voltage, 65 V. MS and MS/MS spectra were both collected at a rate of 1.0 spectrum per second with a medium isolation window of 4 *m*/*z*. The collision energy for MS/MS was varied from 5 to 35 V. An ion mobility mass spectrometer (SYNAPT HDMS, Waters) was used to confirm several metabolites under identical chromatographic conditions.

### 4.9. High-Throughput RNA-Sequencing (RNA-Seq) and Bioinformatics Analysis

Total RNA was isolated from kidney tissues using TRIzol reagent (Invitrogen, Waltham, MA, USA), according to the manufacturer’s protocol. A NanoDrop spectrophotometer (Thermo Fisher Scientific, Waltham, MA, USA) and an Agilent 2100 Bioanalyzer (Agilent Technologies, Santa Clara, CA, USA) were used to evaluate the quantity and integrity of the extracted RNA. The paired-end sequencing approach was used to sequence the samples on an Illumina sequencing platform (Illumina, San Diego, CA, USA). The sequencing data were then mapped to the reference genome (*Mus musculus*; GRCm38) using CLC Genomics Workbench v9.5 software (CLC bio, Aarhus, Denmark). The reads per kilobase million (RPKM) number was used to calculate gene expression levels. To identify and compare pathways enriched by the differentially expressed genes (DEGs) from the RNA-seq datasets, canonical pathway analyses were performed using ingenuity pathway analysis (IPA) (IPA build 2021-02-18, version 62089861). An integration between transcriptomics and metabolomics was performed using PathVisio software (v3.3.0 (http://www.pathvision.org, accessed on 23 February 2015) to evaluate the visualisation of the biological pathways [13].

### 4.10. Statistical Analysis

All data is presented as a mean ± standard deviation (SE). For multiple-group comparisons, a one-way ANOVA was employed, and an LSD post hoc test was utilised to determine the significance of the differences in group means. The unpaired Student’s t-test was used to examine the histopathological findings. In all analyses, statistical significance was set at *p* < 0.05.

## 5. Conclusions

In conclusion, a urine metabolomics study of pathogenic and nonpathogenic *Leptospira* spp. infections revealed significant differences in the metabolites of tryptophan degradation products, carnitine and acyl-carnitines, and the TCA cycle. By integrating urine metabolomics and kidney tissue transcriptomics, increased urine L-carnitine and acetyl-L-carnitine levels in mice with pathogenic *Leptospira* spp. infection were found to be involved in metabolic pathways such as fatty acid activation, mitochondrial L-carnitine shuttle pathway, and triacylglycerol biosynthesis in renal tissues. This study highlights that L-carnitine and acetyl-L-carnitine are implicated in *Leptospira* infection-induced AKI, suggesting their potential as metabolic modulators.

## Figures and Tables

**Table 1:** The quantitative lesion scores of the kidneys obtained from *Leptospira*-infected mice at day 7 after infection.

Measurements *^a^* Groups			
	Without Infection	Pathogenic *L. interrogans* Infection	Nonpathogenic *L. biflexa* Infection
Inflammatory cell infiltration	1.00 ± 0.89	2.33 ± 1.03	1.00 ± 0.00
Degeneration/necrosis	0.83 ± 0.75	1.67 ± 1.03	0.67 ± 0.58
Tubular dilation	1.33 ± 0.82	2.33 ± 0.82	1.00 ± 0.00
Fibrosis	0.17 ± 0.41	0.67 ± 1.03	0.00 ± 0.00
Total histological score	3.33 ± 2.16	7.00 ± 3.29	2.67 ± 0.58

*^a^*: Values represent mean ± standard deviation. *n* = 6.

## Data Availability

The RNA sequencing data have been deposited in the Gene Expression Omnibus (GEO) under the accession number GSE185382.

## Supplementary Materials

The following supporting information can be downloaded: Table S1: https://figshare.com/s/cf1204a6aed9ad4a4778 (accessed on 5 February 2022), Table S2: https://figshare.com/s/3a3a7cc1a68118e4a671 (accessed on 5 February 2022), Table S3: https://figshare.com/s/d354e903abcf6e91e059 (accessed on 5 February 2022), and Table S4: https://figshare.com/s/729b7919b5516e2a95f2 (accessed on 5 February 2022).
